# Strategies to Reduce the Harmful Effects of Extreme Heat Events: A Four-City Study 

**DOI:** 10.3390/ijerph110201960

**Published:** 2014-02-13

**Authors:** Jalonne L. White-Newsome, Sabrina McCormick, Natalie Sampson, Miatta A. Buxton, Marie S. O’Neill, Carina J. Gronlund, Linda Catalano, Kathryn C. Conlon, Edith A. Parker

**Affiliations:** 1 WE ACT for Environmental Justice, 50 F Street, NW, Ste. 800, Washington, DC 20001, USA; E-Mail: jalonne@weact.org; 2 George Washington University School of Public Health and Health Services, 2100 M Street, NW, suite 203, Washington, DC 20037, USA; E-Mail: sabmc@gwu.edu; 3 Department of Health Behavior Health Education, University of Michigan School of Public Health, 1415 Washington Heights, Ann Arbor, MI 48109, USA; E-Mail: nsampson@umich.edu; 4 Department of Epidemiology, University Of Michigan School Of Public Health, 1415 Washington Heights, Ann Arbor, MI 48109, USA; E-Mails: mabuxton@umich.edu (M.A.B.); marieo@umich.edu (M.S.O.); gronlund@umich.edu (C.J.G.); 5 Department of Sociology, City University of New York-Queens College, 65-30 Kissena Blvd, Flushing, NY 11367, USA; E-Mail: lrc10@earthlink.net; 6 National Center for Atmospheric Research, P.O. Box 3000, Boulder, CO 80307, USA; E-Mail: kconlon@ucar.edu; 7 Department of Community and Behavioral Health, College of Public Health, The University of Iowa, N432A CPHB, 105 River Street, Iowa City, IA 52242, USA; E-Mail: edith-parker@uiowa.edu

**Keywords:** extreme heat events, climate change, urban areas, vulnerable populations, health risks, heat-related health interventions

## Abstract

Extreme heat events (EHEs) are becoming more intense, more frequent and longer lasting in the 21st century. These events can disproportionately impact the health of low-income, minority, and urban populations. To better understand heat-related intervention strategies used by four U.S. cities, we conducted 73 semi-structured interviews with government and non-governmental organization leaders representing public health, general social services, emergency management, meteorology, and the environmental planning sectors in Detroit, MI; New York City, NY; Philadelphia, PA and Phoenix, AZ—cities selected for their diverse demographics, climates, and climate adaptation strategies. We identified activities these leaders used to reduce the harmful effects of heat for residents in their city, as well as the obstacles they faced and the approaches they used to evaluate these efforts. Local leaders provided a description of how local context (e.g., climate, governance and city structure) impacted heat preparedness. Despite the differences among study cities, political will and resource access were critical to driving heat-health related programming. Upon completion of our interviews, we convened leaders in each city to discuss these findings and their ongoing efforts through day-long workshops. Our findings and the recommendations that emerged from these workshops could inform other local or national efforts towards preventing heat-related morbidity and mortality.

## 1. Introduction

Extreme heat events (EHEs) can be defined as summertime weather that is substantially hotter and/or more humid than the normal average for a location at that time of year [1]. The term extreme heat event is often used interchangeably with heat wave. EHEs can have negative impacts on personal health by contributing to heat illnesses such as heat cramps, heat exhaustion, heat stroke, and death [2,3]. Extreme heat events are consistently related to mortality [4]; cardiovascular, respiratory and other underlying diseases [5,6,7,8]. In 2012, heat caused more fatalities than other severe weather events [9]. 

With the projected increases in EHEs from climate change [10], communities are attempting to develop sustainable and effective local adaptation programs that consider the health effects of heat [11]. In many locations, climate change adaptation frameworks, or “*adjustments in the individual groups and institutional behavior in order to reduce society’s vulnerability to climate*”, [12] have been developed to assist decision makers and communities in reducing health risks [13,14,15]. For example, the Climate-Ready States and Cities Initiative of the U.S. Centers for Disease Control and Prevention helps 18 states and cities to predict health impacts, monitor health effects, and identify areas most vulnerable to climate change. New tools have also been developed that allow the general public to offer real-time information to practitioners as they address the needs of diverse populations during climate-related hazards, like EHEs [16]. Consistent with these efforts, researchers continue to assess the effectiveness of heat health warning systems (HHWS) based on local weather forecasts and knowledge of adverse health consequences of heat [17]. However, there is currently limited understanding of how local governments use these data, tools and other strategies in preparation for impending EHEs and during these events [18]. 

As part of a larger study exploring heat-related interventions and behaviors, we examined the strategies and methods used by four American cities to reduce the harmful effects of EHEs, how the differences in local context might have affected these efforts, and the common obstacles they faced in this process. Our findings also include indicators for evaluating these strategies and possible next steps, some of which may be generalizable beyond our four study cities. 

## 2. Methods

We conducted 73 in-depth, semi-structured qualitative interviews with officials from government organizations (GO) and non-governmental organizational (NGO) leaders in four cities: Detroit, MI; New York City, NY; Philadelphia, PA and Phoenix, AZ. These cities were selected based on the following criteria: (1) to represent a continuum of implementation of evidence-based heat and climate change interventions and activities (*i.e.*, some of these cities have implemented evidence-based approaches for a decade or more, others have utilized almost no interventions); (2) to represent diversity in context, demographics and climate, and (3) pre-existing relationships with key GO and NGO representatives to initiate our interview pool. 

To best identify potential community-level interviewees that would represent diversity of the study cities in terms of demographics, geography and vulnerability, we created maps using Geographic Information Systems (GIS) with key census variables. Specifically, we created census-tract level maps of the following variable percentages: Black, Hispanic, White and households over 65 years of age. Because race and age are known risk factors for heat related health concerns, understanding the clusters of these communities would allow us to target the most vulnerable groups as a part of our study. The maps included race percentages as well as percentage of households over the age of 65. We also considered other characteristics relevant to understanding and planning for extreme heat, including the variables shown in Table 1. As the chart shows, the differences between our study cities—particularly the July daily high Heat Index (ranges from 28 in Detroit, to 40 in Phoenix), the number of heat related deaths (ranging from 14 in Detroit to 300 in Phoenix), and poverty levels are factors that might motivate varying degrees of heat-related intervention programming in each city.

Interviewees for this study included organizational leaders from both and GOs (*n* = 52) and NGOs (*n* = 21) who were involved in a variety of sectors, including public health, social services, emergency preparedness, energy management, and meteorology. These interviews were conducted from March 2009 to February 2010. We used purposive snowball sampling where initial participants were selected based on our knowledge of their role in local heat preparedness, and these participants identified further contacts with relevant knowledge and experience [19]. We conducted a total of 22 interviews in Detroit, MI (15 GOs, seven NGO leaders); 17 in New York, NY (10 GOs, seven NGOs); nine in Philadelphia, PA (eight GOs, one NGO); and 25 in Phoenix, AZ (19 GOs, six NGOs). A diverse set of organizations were interviewed. We interviewed individuals in multiple city and county departments, including departments addressing emergency management, public health and aging as well as local National Weather Service officials. We also interviewed individuals from a wide variety of social services, including local branches of the American Red Cross and local Area Agencies on Aging as well as NGOs addressing weatherization and energy needs, homeless populations and specific neighborhoods within each city.

**Table 1:** Demographic, climatologic and epidemiologic characteristics of the study cities.

	Detroit, MI	New York, NY	Philadelphia, PA	Phoenix, AZ
Population, 2010 ^a^	713,777	8,175,136	1,526,006	1,447,552
Population Density, 2010 (persons per square km) ^a^	13,324	69,962	29,473	7,246
Percent Non-Hispanic White, 2010 ^a^	7.8%	33.3%	36.9%	46.5%
Percent Below Poverty Level, 2008–2012 ^a^	38.1%	19.9%	26.2%	21.8%
July daily high HI ^b^ (°C), 1980–2010	28	29	31	40
January daily low HI ^b^ (°C), 1980–2010	−5	−1	−1	8
Number of heat deaths ^c^, 1999–2010	14	125	41	300
Web link to heat related city information	[20]	[21]	Office of Emergency Management, City of Philadelphia [22].	City of Phoenix, Human Services [23].
Number of Interviewees	22	17	9	25

^a^ U.S. Census Bureau [24]; ^b^ HI: heat index, or composite measure of ambient temperature and humidity [25]; ^c^ International Classification of Diseases Revision 10 code X30: Exposure to excessive natural heat [26], this likely underestimates the number of deaths attributable to heat, as other causes of death, such as those coded as cardiovascular or respiratory, are often associated with high ambient temperatures [27].

Participants were asked to describe heat-related programming, collaborations, program reach, messaging, and program evaluation during interviews that lasted approximately one hour. Interview questions were developed based on specific research team members’ previous experience and interaction through heat-related research projects in some of our study cities. The research team also conducted an extensive literature review of the heat-epidemiological literature, discovering many gaps and unanswered questions that we used to frame our interview questions and to probe interviewees in cases where initial interview questions were insufficient. These questions focused on development and uptake of heat interventions, feasibility in terms of cost and resources, fidelity of intervention, implementation compared to the prototypical model, what made it difficult or easy to adapt the intervention, how the adaptation has worked in different populations, and the perceived effectiveness of the interventions in reducing adverse effects from heat waves. For those agencies with limited heat interventions, we focused questions on why there has been limited implementation of evidence-based interventions and some of the barriers that prevented full implementation.

All interviews were transcribed and interviews conducted in Spanish were translated and transcribed as well. Using information gleaned from literature reviews, research and interview questions, a code list was developed. Each research team member used an open and focused coding process [28] to apply codes to each of the interview transcripts. Through an iterative, team-based process, the team checked generally for inter-rater reliability (*i.e.*, that all codes were being applied in the same way). In the process, researchers found data, as expected, related to program planning, epidemiological case definitions, and health behaviors, confirming preliminary codes such as “personal reaction to heat”, “definition of heat wave” and “obstacles to staying cool”. Researchers also found data related to program planning, epidemiological case definitions, and health behaviors, confirming preliminary codes such as “personal reaction to heat”, “definition of heat wave” and “obstacles to staying cool”. 

Team members identified themes *in vivo*. In vivo concepts, for instance, included “compared to the 1995 Chicago heat wave” and discussion of “preventing utility shut-off policies”. This coding process led to the development of approximately 100 codes, which were the basis of a final codebook in which each code had a standard definition. Final codes were then entered into NVivo 8.0 qualitative data management software package (QSR International, Doncaster, Victoria, Australia) and used for focused coding of all 159 transcripts by four members of the research team. Seventy-three of the transcripts were utilized for this analysis, focusing on three themes: Program Reach, Challenges and Evaluation. 

Upon completion of the interviews, day-long workshops in each of the study cities were conducted between December 2010 and July 2011 to share preliminary results and discuss key findings with GOs and NGOS. A summary of recommendations from each workshop was compiled and these summaries are presented in Table 2.

**Table 2:** Summary of workshop recommendations.

City	Recommendations
Detroit	Revisit framing of heat warnings
	Invest in full scale public relations campaign to educate residents on heat and health
	Educate grade school students about climate change
	Ensure that county summer campaign includes a heat health component
	Develop messages that connect climate change to everyday life
New York	Identify strategies to prevent oversaturation of messaging (e.g., home-based care providers have many health messages to deliver)
	Using focus groups, determine how and where to best promote cooling centers to a greater diversity of vulnerable persons
	Make health messages that apply to everyone
	Consider additional risk factors in messaging, such as obesity and risk aversion
Philadelphia	Revisit messaging about where to go (e.g., ride public transportation, cooling centers, mall) during heat waves
	Educate people to participate in traditional cooling behaviors
	Increase messaging to encourage buddy systems or checking on loved ones
	Consider use of social media or partnerships with GenPhilly (http://www.genphilly.org) to remind younger generations to check on vulnerable family members
Phoenix	Create clearinghouse of projects and materials
	Develop “check on your neighbor” programs or messaging
	Work with Salvation Army on trainings for social service providers
	Improve collective definitions of heat wave
	Partner with academics to better translate study findings

## 3. Results and Discussion

In the following sections, we describe the local context and heat-related strategies for each of our study cities. We then highlight similarities and differences across these sites, common obstacles to executing heat programming, and an analysis of the role of evaluation in the overall process of local heat preparedness. We conclude with recommendations for next steps raised in the stakeholder workshops held in each of the study cities. 

### 3.1. Activities and Programming to Address Heat by Location

#### 3.1.1. Detroit, Michigan

At the time of this study, the city of Detroit had a population over 700,000, with majority Blacks. While a centralized, formal plan to address heat related program was not in existence, we found that several GO and NGOs were engaged in actions to protect the health of Detroit’s most vulnerable populations: seniors, the homeless and other persons with medical conditions. Detroit had developed an all-hazard plan based on the National Response Framework [29] or unified command structure, which is a structure that brings together the “Incident Commanders” of all major organizations involved in any incident to help coordinate an effective response, while at the same time carrying out their own jurisdictional responsibilities. The City of Detroit’s Health Department houses both the Office of Public Health Emergency Preparedness and Office of Homeland Security and Emergency Management, providing support for all emergency preparedness efforts for natural disasters. The current All-Hazards Plan housed in the Office of Homeland Security includes appendices useful for heat planning for the City of Detroit, such as plans to address special needs and other vulnerable populations, animals, and details needed for a mass care event (which would include transportation, evacuation, housing, feeding and other health services).

In terms of heat health preparedness, in 2008 Detroit developed a HHWS. The purpose of the HHWS was to create city-specific algorithms, based on local climatological and health data, to predict and help guide when the local forecasting office to issue heat warnings, or trigger EHE-related activities. After the local forecasting office used the system in 2008 and 2009, officials felt there were few funds to develop a further index to improve heat messaging. 

While official tracking of health statistics for HHWS’s is a challenge, GOs used a messaging system called Smart Message along with the web-based Michigan Health Alerts Network (MI-HAN) that alerted public health practitioners with surveillance messages such as, “X number of people in this jurisdiction have died—please refer to the website for more information.” This technology, along with GIS was also used to provide a visual representation of real-time information about cooling center locations and other relevant resources during EHEs to the public and GOs.

Media outlets—primarily television and radio—were commonly used sources to publicize heat messaging to the public. Networked information public hotlines and other pathways, like the Community Emergency Response Team (CERT) members, were also used. CERT programs are national and partner with federal management organizations (http://www.citizencorps.gov/cert/about.shtm) to help train citizens to prepare and respond to emergencies and other natural disasters. These trainings often happen before an EHE event takes place, thus enhancing awareness that heat can pose risks to health. As one interviewee stated:

*My role also is to train volunteers in how to be prepared. I do what’s called CERT. Part of that training is that I always try to include seasonal information*, *so if we’re doing it in the summertime it’s about hot weather*… *We also train other CERT volunteers to go out to the public and hand out information and talk about that also*.

Interviewees discussed other activities to try and engage the public in prevention.

*We educated the public about not waiting ‘til your utilities get turned off to seek assistance from the energy provider*; *spearheading campaign’s like* “*It’s Cool to be Prepared*”, *where we surveyed the general public at large summertime events about their knowledge of heat health risks*; *connecting with established programs*, *like Meals on Wheels*, *to get messages to vulnerable populations*.

On the policy side, several GOs discussed efforts at the state level to bring attention to EHEs. For example, the Governor of Michigan declared the last Wednesday of the month of May to be “Heat Advisory Day” to promote heat awareness. The state, working with the local weather service engaged in EHE messaging through press packets and public information statements sent across wire services. Webpage postings also contained heat safety tips, all often underscoring the concern about heat hazards in Michigan. Local weather service interviewees also talked about the ways that they share important information with the public by conducting weather safety talks to schools and other civic organizations. Other GOs set up key partnerships to expand the reach of the message.

*Through our office*, *we partnered with the Wayne County Senior Services because these fact sheets were geared towards the aging adult*… *we distributed this information to our seniors through the Meals on Wheels program*… *and we also very willingly partner with other agencies*.

Detroit has made special efforts to reach populations that could be most vulnerable to heat. Interviewees mentioned “in-the-field” programs like the Gatekeeper Program where utility service providers go out into the field to look at sites and provide assistance to customers (*i.e.*, senior citizens). If they saw a senior in danger, it was reported back to the Detroit Area Agency on Aging. Other NGOs were created in the last 4 or 5 years to develop processes to help homeless and new immigrants with the heat and cold, mostly in the Southwest Detroit, which has a predominantly Hispanic community. 

Interviewees were also concerned about “obese young people in the Hispanic section of town” and “limitations to using the cooling centers.” As one interviewee said, “they won’t come. They’ll say it’s just for old people or I’ll be ID’d.”

Interviewees talked about the populations that need to shelter in place. Detroit Homeland Security and the Emergency Medics have created a list of people who cannot be moved for heat and other emergencies, and briefly described the benefits of the shelter-in-place practice.

*If you can keep them in one place*, *it’s much better*. *It’s much better if you have a senior citizen place with 300 people living there. If somehow you can keep them cool or warm*, *which ever they need*, *and feed them there than it is to take them to a community center where they*—*where they try to sleep on a cot*. *The Red Cross are good at bringing cots and that but you get some of these people down on a cot*, *you’re not going to get them back up, you know*, *I mean, and so it’s better to shelter in place if at all possible*. *And if it’s a*—*if it’s a small location that’s without power or that, you’re better off to leave them in their home*.

Interviewees also expressed that about whether or not vulnerable populations might have internet access and the release of public service announcements by state and/or local emergency websites. One interviewee said that in the future, “heat-related health information should be passed out during summer school and some policy or plan needs to be put in place to deal with heat hazard situations in public schools.” 

#### 3.1.2. New York, New York

New York City is much larger than Detroit, with an estimated and very diverse population of 8.3 million people. Unlike Detroit, New York City had some heat preparedness systems in place, including discussed a syndromic surveillance system that detected 125 deaths with an underlying cause of “heat” from 1999–2010 (See Table 1) This system gathered these data and other health information “real time” by documenting “heat related EMS dispatches” and gathering “other emergency department data from the triage logs.”

*Over the years*, *since 9/11 really*, *a system has been developed by the Communicable Disease Bureau to process text*, *and pull out syndromes that*, *based on words*, *parts of words*, *phrases*; *and*, *there was no heat syndrome previously*. *We developed an algorithm for pulling heat syndrome from the emergency department (ED)*. *It’s a very small proportion of the heat illness burden*, *but it’s very specific for heat*. *It’s less noise*.

In addition to gathering ED data, the city used a unified command system to organize around emergencies and an HHWS to guide public messaging about heat warnings. When the weather forecasts predicted EHEs, conversations with the National Weather Service (NWS) inspired heat preparedness. As one local official said:

*If there’s a forecast of a heat emergency*, *immediately they will have a conference call involving people of all the agencies that would be involved in meeting needs of people during that heat emergency*, *and the National Weather Service is always on the call*. *And during that conference call*, *we determine as a group how we’re going to respond to the heat emergency*. *We determine*, [*for example if*] *tomorrow at nine o’clock we open cooling centers*, *they’ll be open for a certain amount of time*. *Then each day during that emergency we have another conference call in which we report back*.

New York City interviewees suggested that resources and supplies aren’t usually a problem in these moments. As one interviewee said, “*the city has phenomenal resources. It’s a matter of knowing where those resources are and how to access them.*” New York GOs discussed preparations before the heat season begins as one way in which they attempt to coordinate resources. These activities included, but were not limited to, getting in touch with social service programs and case managers to remind them to update the lists of their most vulnerable clients (e.g., homebound, those without air conditioning), and reaching out to senior centers and those that work with homebound seniors.

*We send it not only to senior centers but we also have a part that we send to our case* [*management*] *agencies because they have to be aware because of the seniors*, *the seniors that may be homebound*… *they may need to transport their homebound seniors to a cooling center*, *they’re aware of that*, *and they’ve taken the precautions to do that*. *For the senior centers*, *what we do is send them a checklist reminding them of different things they need to do in order to be ready for the cooling season*.

In terms of outreach, youth were enlisted as messengers to help educate the local elderly about heat related health risks, and local hospitals conducted programming to discuss general reminders about the summer season. As one interviewee said:

*We discuss the signs of heat exhaustion, the signs of heat stroke*. *We remind them not to stay home even though it feels like they’re okay*, *they might not be*. *They should let somebody know if they’re home alone*. *Just to try to reinforce every year what some of the signs are for them to be looking at*.

Participants discussed unique partnerships, like that with the United States Postal Service’s Carrier Alert Program. If the postal carrier notices that the person doesn’t pick up mail in three or four days, the post office alerts a list of nearby community based organizations that this person’s mail has accumulated and a community based organization near where the person lives is sent in—or the police—to see if the person is well. Other programs that the city has launched include Notify NYC, which can notify anyone of any type of emergency or disaster in the city. The city has also partnered with the union for doormen, maintenance, and the people who stand at the desk in buildings, to assist with heat related interventions.

*We train them to recognize when a senior begins to fail*, *so that*, *you know*: *Mrs. Smith is doing fine every day then all of a sudden Mrs*. *Smith isn’t coming downstairs anymore*, *Mrs. Smith is asking me to go to the store*—*becomes disoriented*; *Mrs. Smith is becoming dirty*. *You know*, *all of these things*. *So then the doorman will notify the Department for the Aging and we will be in touch with Mrs. Smith or try to get in touch with her and someone to her to assist her*.

Community response teams also help with enlisting public engagement and participation by translating materials and sending out CERT teams. Outreach also involved observing and respecting traditions, and building relationships with many diverse communities to earn trust. Some preparedness activities included more tangible activities, like the installation of air conditioners in senior homes. Income qualifications and a prescribed medical need were necessary to get the air conditioners installed through a partnership between the Health Department and the Department of Aging. 

Cooling centers are usually setup throughout the city in libraries, public housing, the Salvation Army and other public buildings. The City of New York used a mass fax system to gather timely data from cooling centers during a heat event. Installing spray or sprinkler caps on fire hydrants (attached to fire hydrants to control how much water is used for cooling sprays during the summertime) was another measure to mitigate the effects of heat.

… *People open up hydrants here in the city*. *These are all things to help either mitigate the effects of heat*, *or actually prevent* … *a reduction in fire protection*—*to make sure the public safety needs are there*. *So this whole spray cap thing*, *the DEP responding to that*—*when it gets hot*, *people open up the hydrants*, *but it doesn’t necessary effect the public health issues of heat*, *and* … *they found that* … *people open them up anyway*, *so we’ll just give them these spray caps*, *which will reduce the flow of water out of them*.

Larger events to educate GOs and NGOs were developed to discuss the impact of EHEs, including the psychological impacts of crises, particularly heat waves, in older adults.

*My focus involves the mental health is the psychological impact of crises in older adults*, *including heat waves*. *Last year we conducted a symposium addressing the emotional impact*. *We invited over 190 service providers to this symposium to discuss these issues. And part of it was heat wave*. *It was very well attended*. *And based on that outcome and the needs of the participants*, *right now I’m working on developing a toolkit for service providers to address issues of emotional impacts of emergencies*, *including heat waves*, *on older adults*.

Some heat-related activities were a part of New York’s PlaNYC released by Mayor Bloomberg in 2007 that mandated 25 city agencies to prepare the city to “strengthen our economy, combat climate change, and enhance the quality of life for all New Yorkers”.

*The plan is 127 initiatives*. *And many of the initiatives were certainly designed to have multiple benefits*, *and many of the initiatives*, *some of the benefits would be to deal with some of the impacts*, *would help with some of the impacts from extreme heat*… *And so the city is planting a million trees*. *The goal is a million trees by 2017*, *both street trees as well as reforestation projects*. *We have many initiatives on air quality that will lower the overall base line air quality*, *you know*, *pollutant levels in the city*, *which will certainly have impacts when you have extreme heat*.

The Parks Department installed green roofs on some of the recreation centers throughout the city that offer benefits for stormwater, energy, heating and vegetation. New York City’s heat programming was diverse and often focused on engagement. Many GO and NGO interviewees focused on communicating with the public as key to minimizing the health risks of EHEs. They pointed out the importance of using testimonials and real experiences from the ground that underscore the need to prevent negative health impacts. Other, more structural approaches to reduce the urban heat island effect were also critical aspects of City efforts.

#### 3.1.3. Philadelphia, Pennsylvania

The City of Philadelphia has the longest heat-health intervention program in place, and is often considered the gold-standard for EHE preparedness in the US. City leaders described their evolving programming, and need for ongoing evaluation of its effectiveness. Philadelphia’s heat plan was initially written around the summer of 2008 and provided an overview of specific heat impacts on individuals, such as sunburn, sunstroke, dehydration, as well as the impact of larger events on the larger population, like blackouts. The plan delineated agency roles and responsibilities, particularly for the Health Department that takes the lead in identifying the threshold for activating heat emergencies in Philadelphia. Philadelphia housed the first HHWS, which used city-specific data and an algorithm to provide output for local forecasters to determine whether or not to issue a heat advisory, warning or watch. GOs pay very close attention to the NWS’ warning to make these decisions. As one official described:

*The NWS runs this program throughout the summer*. *They contact me when they see something on the horizon and often that is an internal heads-up and then it’s my role to get in touch with some of the agencies that need that information earlier*… *The ones that really need it earlier are the Corporation for Aging because they’re going to staff the heat line*, *and the Fire Department because they are going to put on extra books because they run EMS*, *Emergency Medical Service*. *Then when we actually have a warning*, *I contact PCA (Philadelphia Corporation for Aging) and I contact our own Environmental Health Services Unit here*. *They get to work on activating the heat line*. *It sort of all happens simultaneously because at the same time this is going on*, *I contact the mayor’s office to make them aware of the contact*, *the managing director’s office*, *contact other folks in the health commissioner’s office who need to know about it*. *Put a call into the water department to stop them from terminating service because they’re non-payment*. *I call PECO, the electric company*, *for the same purpose*. *I call and it actually says on this sheet OESS*, *but it now has a new name*, *OSH (Office of Special Housing)*.

Communicating during a heat event is critical to implementing components of the heat plan. Philly 3-1-1 is a business-hour hotline helpline operated by the Pennsylvania Commission on Aging that becomes a “heatline” in a heat emergency (*i.e.*, hours are extended beyond the typical hours of the business hotline). GOs staff the heatline, provide general information, and also determine whether a call might need to be directed to a medical professional. If so, a public health nurse on staff talked to the individual to determine whether or not an intervention was necessary, and, when necessary, a sanitarian was sent out to the caller’s home. In some cases, the attending nurse provided assistance as in the following case:

*We get calls from people out in the counties asking* “*What should we do*? *I’m really hot*. *I don’t have air conditioning*. *This is a heat wave*. *It’s been hot for a couple of days*. *What am I going to do*?” *And I remember talking to one older woman who couldn’t cool down*. *And I suggested that she get out to the library or the senior center in the daytime if she could*. *But I said*, “*If it’s really hot and still at night*, *what does your basement look like*?” *And she said*, “*Actually I have a family room down there*.” *And I said*, “*Is there a couch*?” *I said*, “*Can you get down there safely*?” *And I said*, “*It’s usually as much as twenty degrees cooler in the basement*”, *from the third floor*, *where her bedroom was*. *So I said*, “*You’d be better off moving down there if it’s safe for you to get down there*. *And she did. And so that was fine.*”

Other activities, like fans and air conditioner distribution programs, were offered to elderly, low income people through the Low Income Home Energy Assistant Program (LIHEAP). The Fire Department and Water Department also conducted assessments in different neighborhoods to assess risk and response needs. Interviewees discussed other measures like distributing fans and operating cooling centers, using lessons learned from larger cities.

*The Deputy Director of Emergency Management used to work in New York’s OEM*, *in the planning division*, *and she’s taken some lessons learned*, *what to identify*. *And so what we’re doing with this cooling strategy is that we’ve now decided*, *we get the notifications from the Health Department every time there’s going to be a heat emergency and use that as the threshold for activating the cooling center strategy*… *the notification will go out to other agencies that have been identified as cooling sites*…

While this strategy was fairly comprehensive, resource limitations made it difficult for officials at the Philadelphia’s Office of Emergency Management to continually update the website to reflect the status of 53 library branches functioning as cooling centers. All cooling center numbers were posted on the website and encouraged residents to call a cooling center before they go. 

NGOs have also worked to continue to raise community awareness around the heat program through organized block/floor captain programs and other initiatives.

*What we say in our public message is that we urge the public to check on older friends*, *neighbors*, *and relatives*, *and the Streets Department has a block captains’ program*. *We talked to the block captains program and we asked the block captains to check in neighborhoods when a heat warning is declared*. *And I have to say that isn’t an entirely reliable system*, *but it’s what exists and I’m sure it does help*, *not in every block*… *block captains check on their elderly residents on their blocks*. *So it really has gotten into the city culture that heat waves are dangerous and here’s the response to them*.

Local leaders also described brochures that contain recommendations for emergency preparedness, especially for what to do in extreme heat including wearing layers and staying out of the sun. These brochures were provided in seven different languages because of diverse populations with low English proficiency. Planners also coordinated with the faith-based community because “a lot of the vulnerable populations were very heavily concentrated in particular areas where they have churches, so we reach out to churches.” Based on previous experience, a community leader in Philadelphia discussed the importance of a pre-EHE strategy of building collaborations with faith leaders to communicate multiple health risks to communities in several cities. That community leader observed:

*We found through experience that developing a relationship with faith and community leaders are the most effective ways of having people trust in the directives that the government is giving them*—*by having this information communicated through religious and faith leaders*.

A respondent from Philadelphia also discussed the importance of targeting messaging to reach populations who might be harder to reach and more at risk.

*We try to get the message out for anybody, really, through the media*…*and say that if they’re elderly you should check on people. If you’re elderly you should have somebody check on you.* …*and if you’re on medications, you know, if you’re at high risk, children, don’t stay outside, and don’t engage in any rigorous activities during the high heat periods.*

GOs and NGOs made special efforts to reach isolated populations.

*We’re doing a lot of things now to find ways to reach an established relationship with communities don’t rely on mainstream media*…*We went around, and this was prior to Katrina, we talked to the Corporation of Aging and asked them what their capability was in terms of reaching people in an emergency. They agreed to partner with us in any sort of catastrophe*…*People who were once institutionalized—and once easy to find—are now being placed in neighborhood-based housing. They’re not in institutions. They’re in communities. They’re harder to find. They agreed to partner with us so we could get to them. Likewise, we looked at what are the networks in place to get information to people with drug and alcohol problems. We talked to the homeless office*… *What we’re looking at right now, we actually have the first one on the table, and we are looking at issuing a health bulletin on pretty much a quarterly basis to distribute to community groups and agencies that we want to establish a relationship with. It also is probably going to include daycare centers, boarding homes, groups that represent different ethnic groups*, *community associations*…

GOs also implemented home-based outreach, specifically in homes where residents received assistance from agencies and NGOs. Environmental health staff went out to check personal care homes to assess if the homes are in compliance with the requirement that all common areas of these homes maintain a temperature no greater than 81° F (27.2 °C) PCA also sent teams to resident’s homes to assess safety, and uncovered other potentially dangerous behaviors.

*A lot of older people that don’t have air conditioning shut all their windows thinking that they don’t want to let the heat in, but what they’re doing is creating this convection oven kind of effect. So they’ll (PCA) will go to houses and distribute fans.*

In addition to intervention activities in response to a heat event, local leaders were working to develop longer lasting interventions to reduce the urban heat effect through energy efficiency projects, specifically focused on cool roofs.

*We passed legislation this year to integrate a cool room provision into our building code so that all new construction in the city—both residential and commercial—will require cool roofs, which is an intervention that again we want to try to start thinking about how we can make impacts at scale. All buildings that have since gone for a building permit had to comply with that regulation. And what we’re hearing, you know, other cities have done this as well*… *And we’ve been talking a lot with the contractor community who say that this has really become common practice because a cool roof has multiple benefits. It has the environmental benefit. It reduces energy use in a house and helps moderate temperature.*

The city partnered with the Dow Chemical Company and the Energy Coordinating Agency, a non-profit in Philadelphia that does energy efficiency improvements, to launch a program called ‘Retrofit Philly Program’.

*They conducted a citywide contest where an entire city block could come together and apply to have their block be the coolest block in Philadelphia. And the winning block, every house on the block got energy efficiency, they got energy efficiency sealing and coating and a white roof on their house. Yeah, so it, we had a tremendous response. It required neighbors to talk to neighbors and to put an application together and to write an essay, so on and so forth. And we had about eighty blocks from across the city participate. And a block can be 100 different homeowners. So getting all those people talking to each other was pretty amazing.*

The city was expanding its green initiatives for sustainability, including planting trees and increasing urban canopy, despite financial challenges.

*We’ve had a number of budget constraints that have eaten into the program budget for our tree campaign. But we’re still committed to that number* [*planting 300,000 trees throughout Philadelphia*]*. And something that we really talk about is the benefits of trees and part of kind of the conversation we’re trying to have as a city and the justification that we want to get out there for funding for trees is the public health benefit. That there’s physical well-being benefits, there’s public safety benefits. But when it comes to the urban heat island effect, we know that trees have a positive impact on their environment and reduce, improve health in extreme heat events.*

The city was continuing to make changes to the building code to improve energy efficiency requirements for new construction. Because Philadelphia is a very old city with little new construction, retrofitting buildings is a challenge.

*What we came to understand in our context here is that there were few opportunities for business owners and homeowners to access lending products to get loans to make energy efficiency improvements. So you can go get a home equity loan to fix up your kitchen but you can’t get an equivalent energy efficiency loan*… *so we were able to get some funding to develop a loan product… One is called the Green Works Loan Fund. Those are large loans for industrial and commercial buildings. And then a loan product for homeowners that’s a version of Pennsylvania’s Keystone Help Loan. So what we were realizing is that people, a lot of people at least understand that there’s benefits to energy efficiency, but they need help taking action. And we’re totally dependent on, you know, we can’t subsidize retrofits. We get weatherization money. We can only do a couple thousand units a year. So in order to do that to start dealing with our infrastructure, our building stock at scale, we needed the private market to kind of step in and start doing something and self-financing. So we also, so we put out the commercial lending and the residential lending program. That’s going to be a huge focus for us and the next two years and beyond is to really understand how we can motivate both businesses and homeowners in Philadelphia to make energy efficiency improvements.*

Philadelphia’s heat programming has been planned longer than most cities and involves a diversity of approaches which depend on local citizens and organizations to engage with vulnerable populations. While the City uses traditional approaches like mainstream media, it also attempts to use innovative communication and engagement with groups that are vulnerable to heat, but who often go unaddressed in response measures.

#### 3.1.4. Phoenix, Arizona

Of the four cities in the study, Phoenix has the most days of extreme heat. GOs and NGOs have several activities around EHEs. Phoenix had a heat emergency plan in place and a HHWS to support heat warning forecasts and messages. In terms of communication with the public, the city had several activities underway. The Maricopa County Health Department used email to communicate directly with case managers assigned to agencies that care for the mentally ill, encouraging them to make sure clients stay hydrated during EHEs, as well as maintain constant communication with first responders, stressing the need to respond to the most vulnerable populations (e.g., elderly) for heat-related incidents. For example, an emergency management communication tool sends out text messages related to heat during the summer that might say, “It’s been 56 days over 100 °F (37.7 °C)” because of the increasing vulnerability that tracks with longer periods of heat. Facilities licensed through state agencies in Phoenix, such as assisted living facilities and group homes, would also be contacted. Another strategy s billboards placed in the airport to communicate heat risks and the importance of staying hydrated to “out-of-towners”. The city’s public health information campaign also started before the heat began.

*The city does a public service announcement on what to do in the heat and watch out for your neighbor and signs of heat stress, and dehydration and kinds of things. So there is whole public campaign. And then we really emphasize it at our senior centers so with the 15 senior centers we do a whole public information campaign. So beginning of the summer we you know the big thing with the seniors is watch out for your neighbors so you know if you don’t see your neighbor. So we do the whole thing on heat stress and education on what you can do and stay hydrated and all that*.

The city Phoenix partnered with the State Department of Education’s established heat education program called SunWise, where educators talk to students about sun exposure. This program reached about 1,000 schools, targeting youth as “the greatest amount of sun exposure to a person tends to be from the time they are born to 18 years of age”. In addition to children, officials talked about educating other vulnerable populations.

*We try to get information through our Behavioral Health Services because people that might be on certain medications are more susceptible to heat-related illnesses or more severity in the illness, we try to get the messaging out to all the people that might be working with folks that might be on these kinds of drugs so that they tell them to watch their sun exposure as best possible; that kind of thing*… *So we try to do a lot of informational things through our licensing capabilities, through our partnerships with providers of services as well as local community towns, cities, counties type things.*

The local United Way convened a county-wide task force. This coalition was made up of relief services that started meeting because of heat related issues and now meets on a monthly basis, year round.

*We spent about four years working together really trying to educate ourselves as to what are the issues and what are ways to mitigate urban heat island and how can we, you know, sort of change the culture, because, you know, you always have to sell inside before you can get it to the outside where you can see the private sector do it*.

The city also provided water for hydration, through donations from public and private entities.

*We have warehousing of water donation. That is one of the things that we do is our city through our mayor and some of our corporate leaders that are in the private sector began a cause in April of trying to get commercial donation of water very, very early*… *we had donations two years ago from a water company in California that heard it was so unseasonable warm and we were having 33 days unfortunately here is how it works. Donations for water don’t usually come until we had the very first significantly hot day*

One of the main relief agencies was the Neighborhood Services Department. One of the programs they provided worked with those residents who are on a fixed income and needed air conditioning in their homes. The program is offered to around 40,000 residents and they typically receive over 100,000 requests, which leaves a portion of the population without AC. Regardless, the city has established a large network of hydration stations and refuge locations that can serve the same function—to provide a cool space for residents.

*This summer there were 70 to 76 hydration stations and refuge locations all across the county where people can go simply to get water or for refuge to get out of the heat and get water, and we collect all sorts of other donations as well like sunblock and hats and t-shirts and flip-flops*… *for the most part, they collect all that information and then provide it to me for the map. And then for the other cities and towns in the region, I collect that information from them and kind of do calls out to the community to see who would like to be involved, and then collect on that information, and then map the locations and then we distribute those to the community*.

There are specific cooling centers for the homeless population as well. At a minimum, these sites were “a cool place where someone can come and sit down or lay down”, some provided food or clothing and in some cases, showers.

*There have been watering and cooling stations set up all over the county, in places where the homeless congregate. And there has been a big drive to collect water and distribute it among people who are homeless or outside, you know, or whatever, and that’s been managed by a lot of homeless organizations, shelters and public healthcare for the homeless clinic*.

GOs and NGOs engaged in a series of outreach and volunteer programs that target homeless populations that may live in the surrounding desert camps.

*We do a lot of outreach*… *we are driving vans out in the desert in the washes, river bottom and places where they actually setup camps. And so we will get temperature sometimes in the excess 115 degrees and so what we do is load up vans with ice chest with water, Gatorade and hygiene kits, socks, underwear and I run a whole group of volunteers from the community that go out with my case manager and our sole purpose in the summer is to hydrate. So I have a deal with PepsiCo and they give us free Gatorade so you know we really try to pump that Gatorade out there and the water.*

Police officers and other volunteers constantly patrol the streets looking for people who are suffering from effects of the heat. Police officers carry water in their police cars, sometimes transporting people in need of medical attention to a local hospital.

*For two years now one thing that they have done is they’ve worked with the outreach teams, and if someone calls in and it's not a dire emergency where they should be calling 911, but they need some sort of assistance and some help, then they will dispatch an outreach team to go to their location and bring them resources, and then if they need to transport them to the emergency room or to a refuge location, then the outreach team will do that*.

GOs note that among the homeless, mental health problems may exacerbate risks of heat illness, thus requiring the assistance of mental health professionals during outreach.

*We have* [*people*] *out there wearing jackets and 3 or 4 layers and it is a 110 degrees out there. So thinking on my team how can I save this person because if I don't get them hydrated and they are too mentally ill to know that this heat is going to kill them. So I want to put a psychiatrist on my outreach team where I can actually if they are willing and they don't want to be hauled off and they don't have to go through court order evaluation and they are willing to take medication, prescribe on the street. And so if we could at least get them on some medication and help get them at least a little bit stable and make the voices go away where we could really engage them and get them treatment and get them off the street. So I am thinking if we had a specialized outreach team that could really meet the needs of those people that are falling through the crack, that would be very helpful.*

Phoenix has also engaged in efforts through the development of task forces to reduce the urban heat island burden.

*I would say the tree and shade task force could be a mitigation strategy because they realized that even for south western cities Phoenix is way under tree canopy average tree canopy. Um there are ... there are greening projects like one group at ASU has canal scape and they are working on how they can reclaim the canals and make those um you know green areas, cool area, recreational areas.*

The City of Phoenix has special environmental considerations in their heat response since it experiences long stretches of heat exposure that affects particular kinds of vulnerable populations, like the homeless. Response measures include communication efforts to all residents and specific response strategies during extremely hot periods. 

### 3.2. Comparing the Activities of Our Study Cities

In the following sections, we outline several important similarities across our study cities, including (1) the development of a formal response strategy, (2) how local factors affect implementation, (3) the focus on addressing vulnerable populations, and (4) the need to accurately track heat morbidity and death. In the next section, we explore obstacles to heat programming, including: (1) financial constraints, (2) cooling center challenges and (3) communication challenges, and (4) the need for evaluation efforts to execute heat programming. 

#### 3.2.1. Similarities across Cities

##### A Formal Response Strategy

Each of the study cities had or were developing a formal plan to address EHEs through programming. The motivation for initiating this type of programming varied, based on political will and resources. In New York, there was significant support from the higher offices of government to integrate heat- and mitigation- related activities within existing or newly forming plans like PlaNYC. However, in other cities, like Detroit, the heat health planning seemed to develop more slowly from a grassroots movement with community members and NGOs making the issue more of a priority for city officials. In Phoenix, heat has always been a concern due to the nature of the climate, thus more organized activities were in place. In Philadelphia, the motivation came directly from the higher incidences of heat related deaths that the city experienced in the 1990s. 

All of the cities had some version of a heat health warning system. The HHWS provided some guidance for warnings, but perceptions on the best use of that information differed across interviewees. For example, local circumstances (a parade or fair), in addition to forecasted weather conditions, would sometimes affect whether a city would activate an outreach program, regardless of the HHWS recommendation. In addition, most of the cities that distributed air conditioners to vulnerable populations found that many in those populations could not afford the electrical costs to operate the ACs. This was cited as a challenge especially by interviewees in New York and Philadelphia. 

Tying heat-related programming with other city efforts on general sustainability and mitigation, seemed to be beneficial, especially for the larger cities. Detroit, Phoenix, and New York integrated heat related program into an existing unified command/emergency response structure. Disregarding city size, this integration was a key factor in ensuring heat was a part of the program. Community engagement was a part of each of our cities activities, however it was more of a force in the smaller, less-resourced cities like Detroit and Philadelphia. All cities had outreach programs that included activities specifically focused on various vulnerable populations. For example, in Phoenix, Detroit, and New York, interviewees mentioned various ways programs deal with those who have psychological disabilities that impede heat adaptation. 

##### Local Factors Affect Implementation

While each of the cities were motivated to approach heat-health planning in some way, a variety of local factors were identified by interviewees that may have influenced how heat programming was implemented. Resources and city structure/organization shape localized possibilities for heat programs. For example, Detroit’s resource challenges like lack of funding and agency consolidations were starkly different from New York City, where resources did not seem to be as much of a concern in our other cities. One of the challenges noted in under-resourced cities, like Detroit, was the ability to continue to update HHWS as needed with new data and other meteorological options to help guide warnings. A city’s climate dictated how and to whom communications were delivered. With Phoenix’s hot and dry climate that is frequented by tourists, leaders had to communicate not only with residents, but also with visitors not accustomed to the local climate. In Philadelphia and New York, it seemed that community members were more open to the messages regarding heat health concerns, possibly due to the past experiences with significant heat waves. However, in a climate like Michigan, many of the messages were focused on priming the audience to understand that heat can be a concern since residents have had limited experience with heat waves. 

Some of our data suggest that, across cities, political will and resources can be critical to driving heat-health related programming in a timely way. If heat-health programming is put forth as a key issue on the “larger political stage”, we are lead to believe that resources from both public and private sources seem to be more available, or people/organizations/companies are more willing to support related programming. However, in cities that are working through financial, administrative and other challenges at the government level, community driven solutions and engagement from NGOs can more than carry the burden and work to address heat health concerns.

##### Addressing Vulnerable Populations

Interviewees in all of the cities seemed to be aware of their vulnerable populations and target the majority of their efforts to reaching these populations. Senior citizens were a constant source of concern. In Phoenix, many activities to reach out to the homeless and tribal communities had taken place. In New York, reaching the diversity of immigrant populations, along with those living in high-rise buildings was the focus. In Philadelphia, those living in row homes were a focus. In Detroit, for example, GOs engaged in an effort to identify those vulnerable to EHE who live in high-rise structures within the city.

*I believe we’ve identified 11 or 12 different high rises in the city where our large clusters of vulnerable populations live. We’ve gone out and actually taken physical pictures of facilities and we’ve made contact with the building managers as well as the people in charge of those housing units. We have identified the units that have air conditioners whether they have central air, for lack of a better term, window shakers, or single unit air conditioners*.

##### Need for Tracking Heat-Related Illness and Death

While the utility of HHWS’s can sometimes be witnessed in a reduction in heat-related morbidity and mortality [30], the tracking of heat-related deaths upon which HHWS’s are based is a challenge. Heat-related morbidity and death are typically under-reported [31], and in some cases, multiple agencies are tasked with tracking these vital heat-related health statistics. As one official said:

*It’s kind of a difficult thing with the Weather Service, we’re supposed to keep track of heat and cold deaths but they’re way under reported*… *The only way that we have access to that is through newspaper or media accounts—or if any emergency manager knows of one death, here or there from heat or cold are not going to make it to the emergency management level. What we have done in the meantime is contacted the state’s Department of Community Health, where the keep the statistics... I find it interesting that is the National Weather Service’s responsibility to keep track of weather related deaths and yet there’s not really a coordinated surveillance system—the way there might be for reporting infectious disease—like CDC is tracking Lyme Disease and what not.*

Interviewees more generally felt that improving the tracking of heat morbidity and mortality could play an important role in triggering heat warnings accurately, and is therefore of importance in adaptation efforts.

#### 3.2.2. Obstacles to Heat Programming

##### Financial Constraints

Financial constraints influenced both the development and implementation of EHE strategies. The primary obstacle to development of strategies was lack of funding. Government officials and community leaders explained that they had received support from local utility assistance organizations, public service commissions, private energy companies, private donations, federal energy assistance programs, local and federal health and emergency preparedness agencies, and federal stimulus monies to carry out programs, but these sources of support were not always available and were often limited. As heat preparedness is relevant to a diversity of sectors, there was concern the issue could get easily “passed around,” without a responsible lead agency at the municipal level to ensure long-term resources, and unforeseen obstacles were overlooked. For instance, interviewees described programs that provided air conditioners to low-income households, later realizing that households could not afford the associated energy bills and did not use the air conditioners. As one city official in Detroit explained, “It looked nice when you put it in, cool. But after a week or so when we drove by, they weren’t using them because once that bill came, they saw and so it’s back to basics again.” Similarly, interviewees discussed how a large proportion of the households most vulnerable to heat could not qualify or were not safe for weatherization programs because their homes needed expensive prerequisite repairs (e.g., new roofing) they could not afford.

Interviewees also discussed how the hypothetical, abstract, and difficult-to-predict nature of heat emergencies affects funding for strategies. They expressed difficulty in securing funds for something that may or may not happen, such as an EHE. Further, justifying such funding by showing that these activities resulted in a decrease in heat-related morbidity and mortality is challenging:

… *if it were tied more to heat-related illnesses and we could say, you know, we could put a sun shelter out there, maybe put some sort of mist system or something like that on them so the temperature is reduced as well*… *I think “if-if-if” a value to it can be demonstrated, other than say administrative type control factors, like controlling the time of day kids are out to begin with, I think it could happen sooner than later.*

##### Cooling Center Challenges

Cooling centers are possibly the most commonly used heat preparedness strategy in cities across the nation for protecting health during heat events, yet many barriers to their use exist. These include: stigma, hygiene, health and safety, access, and the difficulty with evacuating one’s home. Some interviewees spoke of the misconception that cooling centers are only for seniors or homeless individuals, deterring people from going to them. Additionally, cooling centers are intended to prevent heat-related health problems, but they may create a host of new health concerns. For instance, one social service provider described concerns with hygiene in a New York cooling center, leading staff to keep a supply of extra clothes and, in the past, sending clients to the park to shower in fountains before returning. Further, access to centers may be difficult. While many cities have para-transit or public transportation, cooling centers are not always in the direct route of these services. One community center staff member described her client’s experience, which could make him more vulnerable to heat:

… *that bus don’t have no air*… *that is the only thing I hate. Mr. __ is picked up at 9:30 in the morning and* [*the bus driver*] *picks up 8 more people. Mr. ___ is only 5 min*, *10 min from here*… *You could bring him here and go and get the rest of those people, but they don’t do that, and poor Mr. __ is on that bus and in his condition for what, two or three hours before he gets down here, and that to me is cruel. I mean, it has been hot days.*

Finally, as with other types of emergency evacuations, leaving one’s home to go to a cooling center can be emotionally or physically jarring, especially as many people do not know what to expect. One major barrier to this relocation echoed by many interviewees is that, “People are not going to leave their pets, and we saw that with Hurricane Katrina.”

In addition to facing challenges in providing cooling centers, themselves, when cities offered air conditioning units to individual residents, they often faced challenges. Interviewees sometimes noted that deaths among seniors still occurred even if they had air conditioners. As one interviewee said, “they wouldn’t turn it on; either they—it made them uncomfortable, and that does happen, or they couldn’t afford to run it.” 

##### Communication Challenges

Lack of public awareness, providing appropriate evolving messages, and finding ways to share information with those who lack internet access are familiar challenges for many public health issues. An additional challenge for communication about EHEs is that often EHEs are perceived as not as serious or as newsworthy as other natural disasters, which can make it difficult for local organizations and government agencies to start discussions about preventing heat-related health impacts. The phrase “boy who cried wolf” was echoed among government employees to describe their concern that too many warnings of the dangers of heat could result in the public’s desensitization and that the frequency of risk messages could negatively impact the community response. 

Some local leaders noted that even though heat is a leading killer, the results of other types of natural disasters are easier to convey.

*He shows a before and after picture of a tornado, and there is a house. There it is in rubble. Okay, here is a picture of a house, you know, on a normal day, and here is a picture of a house on a hot day. You can’t see the difference*.

Agencies and organizations must consider communication challenges specific to their priority populations. In Detroit, for instance, as one interviewee noted, “Detroit has [nearly] a 50% illiteracy rate…” Cognitive decline associated with aging may also be an obstacle for messaging. One senior interviewee expressed concern that her peers cannot retain messages, “... when a person gets elderly, it’s hard to keep things in the brain. They tell you something today, and say, oh, next week they forget.” 

A New York representative discussed adapting communication to meet the needs of the public.

*I think it’s also how we reach out to the media. we have to take into account those who may be hearing-impaired or blind, because oftentimes … someone may say something but it may not be in the language, or … even for the hearing impaired, going across the bottom of the screen* … *that kind of stuff, that’s not there for them, and I think the city could do better in doing stuff like that.*

One interviewee from Detroit expressed limitations of communicating information over the internet:

*My parents have gotten very good at it. I'm impressed, too. But I think, you know, I think in all of this, though, that we assume that our population is fully accessed to the internet.* …*I know that this website has been pushed with public service announcements in the last few years, but I don’t know if that’s been done recently. Also,* … *I worry that we over estimate the number of people who actually do have access to the internet.*

A continual challenge, as noted by an interviewee from one city, was communicating with two specific isolated populations: people who were once institutionalized but are now in neighborhood-based housing, and the homeless population.

With the variety of strategies and challenges, many innovative opportunities also exist for improving communication of heat’s health risks to urban populations. Compared to our other study cities, Detroit did not have a dedicated website page that provided direct information about their heat plan or other heat information which provides a unique communication challenge. The Detroit Climate Action Collaborative is currently preparing the city’s first Climate Action Plan, which will include strategies for heat preparedness. A government official from Detroit suggested that effective messaging requires understanding the health effects of heat stress.

*When voter registration was coming, we wanted to get people* [*to understand*] *the importance of their vote. We can do major educational campaigns with our client population. We do it all the time. And this probably needs to be one of them. Because*, *for example*, *if somebody is homeless and the heat’s out there*, *we need to be able to educate them*: “*What can I do*, *where can I go when that happens to be the situation*” … *and also to understand the signs and symptoms of when their body’s in trouble because of it*.

Respondents described the opportunity to inform community members about heat risks using educational strategies similar to the door knock programs used for increasing participation in the 2010 U.S. Census and increasing awareness around West Nile virus (a mosquito-borne illness first detected in North America in 1999) [32]. Some interviewees suggested that messages need to focus also on the indirect risks associated with heat. For example, someone with air conditioning might not want to go outside to get his/her medicine, and may become sick or die anyway, not directly due to heat, but from the heat keeping him/her inside.

##### Evaluation of EHE Preparedness Interventions

The obstacles to EHE programming offered opportunities for evaluation. However, despite efforts to identify and quantify the success and reach of heat preparedness programs, the majority of the participants stated that evaluation was difficult, particularly when given limited funding and resources. Interviewees in Philadelphia and Phoenix noted they use surveillance of heat-related deaths, emergency dispatches, hospitalizations and hospital discharges during EHEs to inform future preparedness plans. A representative from the City of Phoenix stated that longitudinal data collection is costly, but necessary, to provide a comprehensive evaluation of heat-related deaths and hospitalizations. Respondents noted the potential utility of conducting best practices assessments and the relevance of comparing heat-related mortality in other cities as a way to boost evaluation efforts.

Interviewees also pointed to the importance of evaluating how their heat programs were being used in order to assess the efficacy of their work. As one interviewee said, “we try to measure as best we can. The mayor, again his background is measuring data. His famous saying to us is, “If you can’t measure it, you can’t manage it.”

Many interviewees noted that heat-related morbidity and mortality are difficult to track, and they commonly used many indirect measures or indicators to inform planning and evaluation efforts. Some interviewees reported using attendance and daily reports at cooling centers to identify the types and quantities of populations visiting and using the centers. However, concern was widespread over whether informal evaluations were adequately capturing the adverse health outcomes of populations most vulnerable to heat. For instance, community centers in New York are used as cooling centers during heat events, making it difficult to tease out whether attendance is simply a routine use of the community center or use of the community center as a cooling center in response to the heat event. 

Other indicators of program success included: the number of individuals requesting and receiving fans in cities with fan distribution programs, and cancellations of City of New York’s Access-A-Ride appointments, a service that provides transportation for disabled individuals, which presumably indicates that elderly and disabled populations were staying indoors in cool environments rather than being “out and about” during EHEs. Additionally, participation in emergency preparedness exercises, as well as requests for trainings and informational presentations on heat-health preparedness, among city agencies and non-profit collaborators were used to illustrate the level of coordination throughout heat emergency service providers.

### 3.3. Recommendations from Stakeholder Workshops in Study Cities

The one-day workshops, in which we shared results with participants and other practitioners in the cities, helped our interpretation of the themes from the interviews and also served as a platform to initiate new collaborations and generate ideas to inform preparedness and risk communication. Table 2 describes some of the suggested strategies gleaned from our stakeholder workshops. 

For example, workshop participants offered reasons for inactivity in EHEs, such as the elderly being concerned about safety that resulted in them not opening windows in EHEs. In these workshops, we also investigated the obstacles to the development and implementation of these activities. Participants in the workshops identified similar obstacles to development and implementation of effective strategies including financial constraints, challenges with cooling centers, communication about the danger of EHEs and evaluation of activities. While workshop participants identified a range of obstacles to effective heat preparedness programs, they also identified pre-existing emergency preparedness partnerships, informal evaluations, and extreme events themselves that help catalyze the implementation of further programs. A challenge also noted in Detroit was the multiple messages from different sources and the importance of coordinating communications through government offices, such as fire, medical, and others.

Workshop participants mentioned several aspects of planning that would bolster strategies and activities to reduce the effects of EHEs. These included cross-agency engagement, communication, evaluation, and integration. For example, stakeholders such as representatives from media outlets, emergency planners, home weatherization organizations, utility companies and other members of private industry, housing authorities, retirement communities, the NWS, organizations representing isolated populations (e.g., the elderly, homebound, homeless), emergency medical services, neighborhood tenant associations, city block captain organizations, faith-based and community organizations should be engaged in these partnership planning activities .

Workshop participants also mentioned the importance of communication efforts across agencies to enhance and improve preparedness. Participants suggested engaging government stakeholders in quarterly lunches or creating newsletters, and using social media to inform them of heat and health work around the city that could promote collaborations across agencies to reduce duplication of efforts. Specific conversations and dialogue with educators, regional decision-makers and experts would also be effective when focusing on related heat issues, such as planning heat adaptive landscapes for cities, environmental justice and water access concerns. This is already occurring in some locations and could work well in advancing preparedness in others. The importance of integrating heat-health planning into existing departmental plans such as emergency response plans and vulnerability assessments was also noted. For example, identifying vulnerabilities during potential power grid failures and transportation-related vulnerabilities (e.g., non-air-conditioned subway platforms, transporting vulnerable populations to cooling centers) would be useful in heat-health planning.

Several approaches to advancing policy regarding heat preparedness were offered during these workshops. First, talking directly with legislators about codifying climate and heat-related programming and plans is an important way to help sustain such efforts during and after changes in local leadership. A second approach is to improve training for health experts and care givers. Interviewees suggested that organizations like the American College of Physicians could provide continuing medical education credits on heat-health related activities. They noted that the National Institute of Environmental Health Sciences could invite research proposals that address both sustainability and prevention for climate change-related health effects. 

Finally, thoughtful integration of heat-health concerns into existing city ordinances and policies on home weatherization is needed. This might include expanding the scope of existing legislation (like the Cool Roofs in Philadelphia) to include health outcomes. Ideally, concerns about heat would be included in new policy measures in order to formalize a commitment to preventing heat-related health problems, both through traditional intervention activities and by improving sustainability of housing and neighborhoods. 

## 4. Conclusions

While population size, poverty level, climate, and level of local experience with heat-related climate planning vary by city and may have shaped activities, participants noted some universal concerns. In particular, results of our research identified what strategies practitioners used for heat preparedness activities to minimize the health impacts of EHEs as well as the challenges and opportunities that exist to implementation. While each of our study cities had many similar activities (e.g., cooling centers, HHWS, fan/air conditioner distribution programs, outreach), the local context (e.g., political will, resources, maturity of community organizations) often dictated the breadth of implementation for various EHE activities. Identifying and reaching the most vulnerable populations (e.g., immigrant communities), collecting and using health statistics effectively (*i.e.*, multiple agencies tracking heat-related deaths, illness—not centralized), and managing resources based on the needs of particular communities (e.g., not opening too many cooling centers) were concerns in all cities. Some of our cities are currently going through major transformations (*i.e.*, fiscally and structurally), making community-driven solutions and engagement from NGOs critical to addressing EHE concerns. The U.S. has experienced economic challenges since the mid-late 2000’s and, as a result, many local governments have increasingly limited resources to dedicate to heat planning. In Detroit, for instance, which is undergoing perhaps the greatest transformation of our study cities, both structurally and fiscally, community-driven solutions may be critical to addressing EHE concerns [33].

Our findings regarding the serious obstacles cities face in addressing heat risks lead to recommendations for advancing programming. The primary obstacles were financial constraints, cooling center challenges and communication issues. Therefore, in order to prevent further heat-related morbidity and mortality, cities should consider dedicating greater resources to heat programming by funding involved agencies specifically to address heat. A first step in this process might be to direct preliminary funding to evaluation of current programming since a number of communication and technological tools are already being used by cities to prepare for EHEs and for outreach or coordination during these events without being evaluated. Using indicators outlined in this paper as well as those of interest locally, these evaluations could help articulate how each location can move forward in preventing heat impacts. For instance, our findings that certain preconceptions and stigma affected community member usage of cooling centers can be used to guide further evaluation and outreach efforts and the selection of cooling centers to ensure improved usage. Finally, the granular findings we have provided here about communication with vulnerable populations can be used by cities to tailor subsequent efforts, especially to populations who are at risk but consider themselves to not be vulnerable. One of the key outcomes of the stakeholder workshops for all the cities was the opportunity for various GOs and NGOs to be in the same room, listen to the common obstacles shared through our interviews, and discuss solutions and next steps, building on some of the themes from the interviewees. For example, in all cities, stakeholders offered recommendations regarding the importance of heat messaging and improving or creating partnerships. 

While the selection of our four cities was aimed at presenting information from cities with geographic and programmatic diversity, some of this information might not be completely transferable to other settings. In addition, while the data collection for this study spanned 2009 and 2010, each of the city specific programs have continued to evolve their heat preparedness efforts and the most current activities might not be captured in this work. Keeping these limitations in mind, our research suggests the importance of the work public health practitioners are already undertaking around EHEs and the need to further explore other potential successful strategies to inform health-promoting policies for cities in the face of climate change. While cities can work toward many of these goals immediately, some of them will need expanded support from external resources such as federal agencies and private stakeholders. 
